# Prognostic Significance of Apparent Diffusion Coefficient in Hepatocellular Carcinoma Patients treated with Stereotactic Ablative Radiotherapy

**DOI:** 10.1038/s41598-019-50503-7

**Published:** 2019-10-02

**Authors:** Cheng-Hsiang Lo, Wen-Yen Huang, Chih-Weim Hsiang, Meei-Shyuan Lee, Chun-Shu Lin, Jen-Fu Yang, Hsian-He Hsu, Wei-Chou Chang

**Affiliations:** 1 Department of Radiation Oncology, Tri-Service General Hospital, National Defense Medical Center, Taipei, Taiwan; 2 Institute of Clinical Medicine, National Yang-Ming University, Taipei, Taiwan; 3 Department of Radiology, Tri-Service General Hospital, National Defense Medical Center, Taipei, Taiwan; 4 School of Public Health, National Defense Medical Center, Taipei, Taiwan

**Keywords:** Cancer imaging, Hepatocellular carcinoma

## Abstract

The role of diffusion-weighted magnetic resonance imaging (DW MRI) in assessing durable tumor control for patients with hepatocellular carcinoma (HCC) treated with stereotactic ablative radiotherapy (SABR) was not defined. This retrospective study included 34 HCC patients with 45 lesions who had DW MRI data at baseline and within 6 months post-SABR. On the first post-SABR MRI, 13 lesions (28.9%) had a complete response (CR), 12 (26.7%) had a partial response (PR), 17 (37.8%) had stable disease, and 3 (6.7%) had progressive disease by modified Response Evaluation Criteria in Solid Tumors (mRECIST). On subsequent imaging, the response rate improved from 55.6% to 75.6%. The apparent diffusion coefficients (ADCs) (mean ± standard deviation) pre- and post-SABR were 1.43 ± 0.28 and 1.72 ± 0.34 (×10^−3^ mm^2^/s), respectively (*p* < 0.001). An ADC change ≥25% (DW[+]) was identified as a predictor of favorable in-field control (IFC) (1-year IFC, 93.3% vs. 50.0% for DW[−], *p* = 0.004), but an mRECIST-based positive response (CR and PR) at the first MRI was not (*p* = 0.130). In conclusion, ADC change on early MRI is closely related to IFC in HCCs treated with SABR. Standardization of the DW MRI protocol, as well as prospective validation studies, are warranted.

## Introduction

Stereotactic ablative radiotherapy (SABR) is a locoregional treatment for patients with unresectable or medically inoperable hepatocellular carcinoma (HCC)^1–3^, and may be used alone or combined with other therapies. With its large ablative radiation dose and precise delivery, the post-SABR response rate can reach 77%; the 1-year local control rate is reportedly 75–100%^1–3^.

Early assessment of treatment response is crucial, and allows for timely salvage or sustained follow-up. The criteria used to evaluate SABR responses in HCCs vary^1–4^; the most preferred systems currently include the modified Response Evaluation Criteria in Solid Tumors (mRECIST) and European Association for the Study of Liver Diseases (EASL), which consider tumor necrosis or non-enhancing components after locoregional therapy^5,6^. However, such enhancement-based criteria have only been evaluated in HCCs treated with non-radiotherapy modalities^7^; their applicability to HCC treatment response evaluation in the early post-SABR phase is unclear.

Diffusion-weighted magnetic resonance imaging (DW MRI) is increasingly used in the detection, diagnosis, and characterization of tumors^8^. Its quantitative parameter, the apparent diffusion coefficient (ADC), reflects the mobility of water molecules within tissue. Tissues with high cell densities tend to exhibit lower ADC values than those with low cell densities, rendering the ADC a surrogate indicator of cellularity. Cellularity change post-treatment can be detected via ADC alteration before tumor size change, and the ADC is a promising predictor of early treatment response in brain, head and neck, prostate, and cervical tumors^9–12^. However, the application of DW MRI in HCC response evaluation after radiotherapy is sparse and undefined, and no studies have focused on post-SABR assessment^13,14^. Hence, we investigated the role of DW MRI in assessing durable tumor control for patients with HCC treated with SABR.

## Results

### Patient characteristics

Thirty-four patients were included in this study; their median age was 65 years (range, 41–85 years), and 29 patients (85.3%) were men. Most patients had underlying viral hepatitis B (55.9%), Child-Turcotte-Pugh class A liver function (76.5%), recurrent disease (73.5%) and Barcelona Clinic Liver Cancer stage C (64.7%). Twenty-six patients (76.5%) had 1 tumor, 6 (17.6%) had 2 tumors, and 2 (5.9%) had ≥3 tumors treated, for a total of 45 tumors. The median tumor size was 3.9 cm (0.8–22 cm); 10 tumors (22.2%) involved portal vein tumor thrombosis. Additional patient and tumor characteristics are summarized in Table 1.

**Table 1:** Patient and tumor characteristics

Variable		n. (%) or median (range)
No. of patients		34 (100)
No. of tumors		45 (100)
Gender	male/female	29 (85.3)/5 (14.7)
Age, years		65 (41–85)
Viral hepatitis	HBV	17 (50.0)
	HCV	6 (17.6)
	both	2 (5.9)
	none	9 (26.5)
Recurrent HCC	yes/no	25 (73.5)/9 (26.5)
Tumor size, cm^*^		3.9 (0.8–22)
Portal vein tumor thrombosis	present/absent present/absent^*^	10 (29.4)/24 (70.6) 10 (22.2)/35 (77.8)
Extrahepatic spread	present/absent	6 (17.6)/28 (82.4)
ECOG performance status	0	15 (44.1)
	1	15 (44.1)
	2	4 (11.8)
AFP level, ng/ml	<200/≥200	24 (70.6)/10 (29.4)
CTP class	A/B	26 (76.5)/8 (23.5)
BCLC stage	A/B/C	9 (26.5)/3 (8.8)/22 (64.7)
Previous treatment	Surgery	9 (26.5)
	RFA	8 (23.5)
	TACE	16 (47.1)
	SABR	3 (8.8)
	Sorafenib	3 (8.8)
	Naïve	9 (26.5)
SABR regimen^*^		
total dose, Gy		45 (30–60)
fraction number		5 (4–6)
EQD2, Gy		71.3 (40–125)

*Abbreviation*: HCC = hepatocellular carcinoma; HBV = hepatitis B virus; HCV = hepatitis C virus; ECOG = Eastern Cooperative Oncology Group; AFP = α-fetoprotein; CTP = Child-Turcotte-Pugh liver function scale; BCLC = Barcelona Clinic Liver Cancer; RFA = radiofrequency ablation; TACE = transarterial chemoembolization; SABR = stereotactic ablative radiotherapy; EQD2 = equivalent dose in 2 Gy fractions.

^*^The data is expressed at the lesion level.

### Tumor response and ADC change after SABR

**Figure 1:**
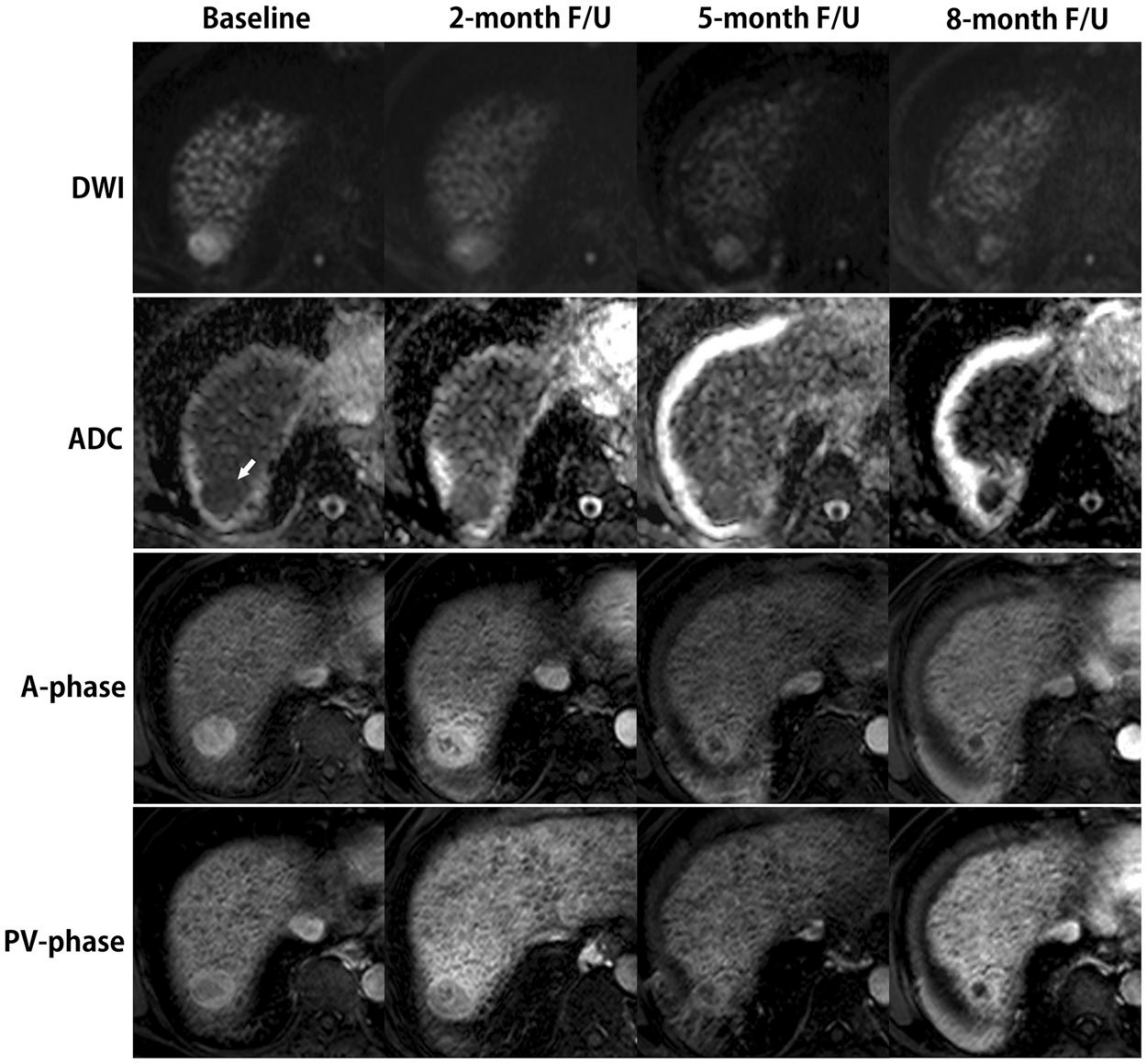
Early ADC change before HCC lesion reduction obtained at baseline and at different times post-SABR. A 71-year-old man with hepatitis B virus-related liver cirrhosis and a 2.8 cm HCC in segment 7. At baseline MRI, the lesion shows strong enhancement in the arterial phase (A-phase) and washout in the portal venous phase (PV-phase), with moderate hyperintensity on DWI (*b* = 500) and an ADC of 1.37 × 10^−3^ mm^2^/s. At the 2-month follow-up, the lesion was stable in size by mRECIST. Lower hyperintensity at DWI was noted with a corresponding ADC of 2.00 × 10^−3^ mm^2^/s (a 46% increase). Geographic parenchymal hyperenhancement was noted in both the A- and PV-phases, consistent with focal liver reaction to SABR. At the 5-month follow-up, the lesion had a lower enhancement size (1.9 cm), indicative of partial response, with resolution of geographic parenchyma hyperenhancement. At the 8-month follow-up, no enhancement was observed, indicating complete response. Continuous volume loss of the overlying liver parenchyma was observed at 5 and 8 months.

The median time between baseline MRI and initiation of SABR was 1.1 months (range, 0.2–2.9 months). The median time between the completion of SABR and first follow-up MRI was 2.3 months (range, 1.0–5.5 months); 28 (82.4%) had MRI conducted within 3 months. On the first post-SABR MRI, complete response (CR) was achieved in 13 lesions (28.9%), partial response (PR) in 12 (26.7%), stable disease (SD) in 17 (37.8%) and PD in 3 (6.7%). The best responses post-SABR were as follows: CR in 21 (46.7%), PR in 13 (28.9%), SD in 8 (17.8%), and PD in 3 (6.7%); of these, 4 PR lesions were upgraded to CR, and 9 SD were upgraded to CR (4) or PR (5) (Fig. 1). The initial PD lesions had no status change at the most recent follow-up.

**Figure 2:**
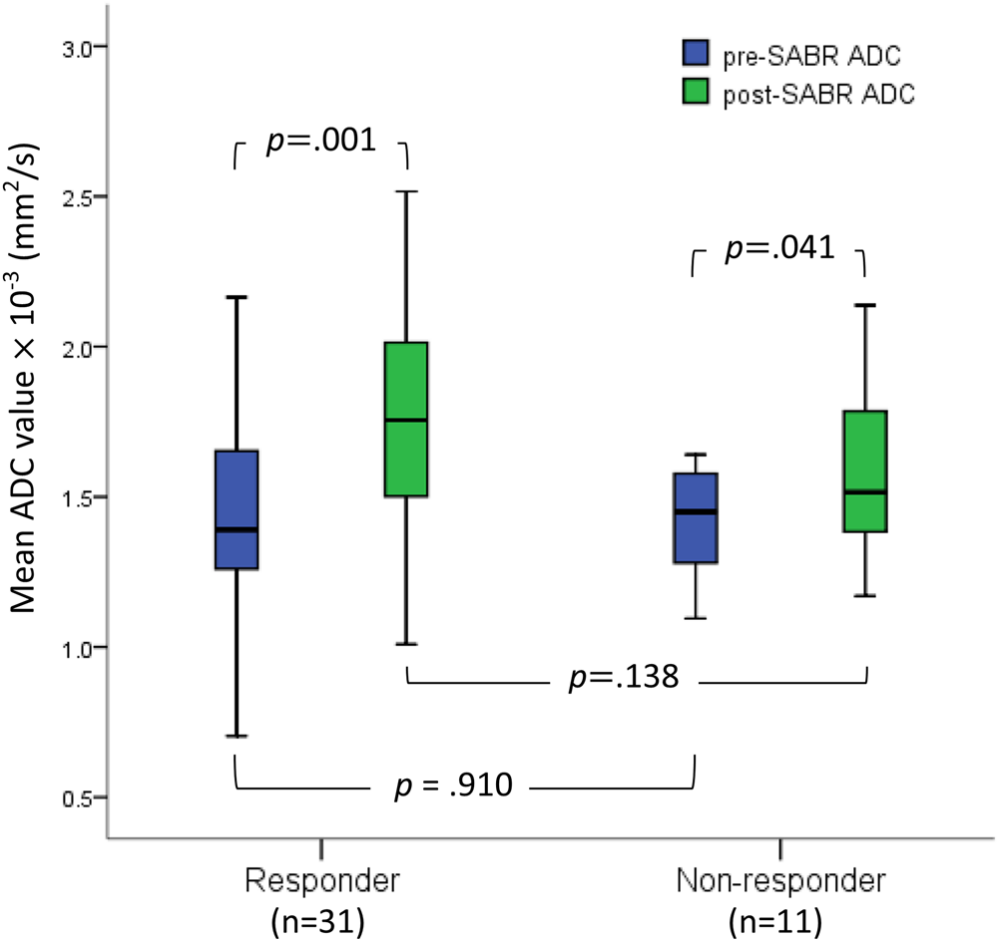
Box-whisker plot of the mean ADCs for responding and non-responding lesions pre- and post-SABR. Three lesions that resolved completely on the first post-SABR DW MRI were not included.

The intraclass correlation coefficient for ADC values pre- and post-SABR were 0.94 and 0.91 respectively, indicating good interobserver agreement; the average ADC value obtained by the observers was used for subsequent analyses. ADC values were measured in all 45 tumors before SABR and in 42 tumors afterwards (the values of 3 lesions that completely resolved on the first post-SABR DW MRI were not measured). ADC values increased significantly after SABR (mean ± standard deviation, pre-SABR: 1.43 ± 0.28 × 10^−3^ mm^2^/s vs. post-SABR: 1.72 ± 0.34 × 10^−3^ mm^2^/s, *p* < 0.001). Significant increases in ADC levels were observed in both responding and non-responding lesions (*p* = 0.001 and *p* = 0.041, respectively) (Fig. 2). No difference existed in pre-SABR ADC values between responding and non-responding lesions (1.44 ± 0.31 × 10^−3^ mm^2^/s vs. 1.41 ± 0.20 × 10^−3^ mm^2^/s, *p* = 0.910). The post-SABR ADC values and mean ADC change percentages for responding and non-responding lesions were 1.77 ± 0.35 × 10^−3^ mm^2^/s vs. 1.59 ± 0.29 × 10^−3^ mm^2^/s (*p* = 0.138) and 27.6 ± 33.6% vs.13.3 ± 16.7% (*p* = 0.149), respectively.

On ROC analysis, the optimal cut-off value of the ADC increment percentage for predicting freedom from in-field failure was 25% (sensitivity = 57.6% and specificity = 88.9%). Accordingly, DW response was defined as an ADC change ≥25% post-SABR; the 3 completely resolved lesions were assumed to have DW responses on IFC analysis.

### In-field control and prognostic factors

**Figure 3:**
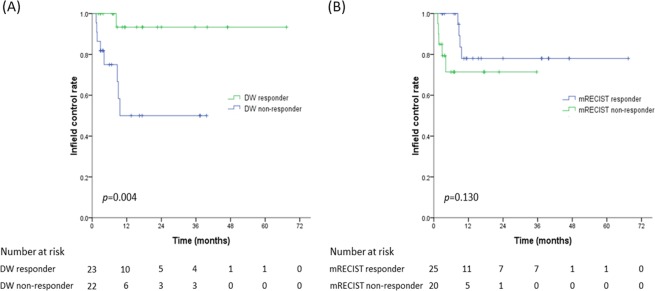
Kaplan-Meier curves of in-field control according to (**A**) ADC response and (**B**) mRECIST response.

The median time to in-field failure for all lesions was not reached; the 1-year IFC rate was 72.9%. Factors associated with improved IFC are shown in Table 2; only tumor size and DW response were identified as predictive of IFC on multivariable analysis (*p* = 0.006 and 0.037, respectively). The median times to in-field failure were not reached for DW responding lesions and 9.6 months for DW non-responding lesions (1-year IFC, 93.3% vs. 50.0%) (*p* = 0.004) (Fig. 3A). The first MRI response did not significantly correlate with IFC; the 1-year IFCs were 78.0% and 71.4% for mRECIST responding and non-responding lesions, respectively (*p* = 0.130) (Fig. 3B).

**Table 2:** Prognostic factors for in-field control by Cox proportional-hazards model.

Variable	Univariable		Multivariable	
	HR (95% CI)	*p*	HR (95% CI)	*p*
Tumor size (1 cm increase)	1.22 (1.07–1.38)	0.002	1.19 (1.05–1.34)	0.006
Portal vein tumor thrombosis	5.79 (1.51–22.3)	0.011		
AFP level, ≥200 vs. <200	3.81 (1.02–14.22)	0.047		
CTP class, A vs. B	3.06 (0.38–24.70)	0.294		
EQD2 (Gy), >71.3 vs. ≤71.3	1.03 (0.28–3.88)	0.963		
1^st^ MRI mRECIST response				
responding lesion	0.37 (0.10–1.41)	0.145		
Pre-SABR ADC (×10^−3^ mm^2^/s)				
>1.45 vs. ≤1.45	1.77 (0.44–7.08)	0.423		
Post-SABR ADC change				
any ADC increment	0.75 (0.16–3.63)	0.723		
≥25% ADC increment	0.09 (0.01–0.72)	0.023	0.11 (0.01–0.88)	0.037

*Abbreviation*: AFP = α-fetoprotein; CTP = Child-Turcotte-Pugh liver function scale; EQD2 = equivalent dose of 2 Gy per fraction; mRECIST = modified Response Evaluation Criteria in Solid Tumors; SABR = stereotactic ablative radiotherapy; ADC = apparent diffusion coefficient.

On first MRI of SD lesions, 9 had a DW response (DW[+] SD) and 8 did not (DW[−] SD); the respective 1-year IFCs were 100% and 65.6% (*p* = 0.127). The IFCs of DW(+) SD lesions were not significantly different from those of mRECIST responding lesions on first MRI (*p* = 0.327), whereas the IFC of DW(−) SD lesions tended to be inferior to that of mRECIST responding lesions (*p* = 0.060).

### Explant pathology and ADC correlation

Five patients with 5 lesions were censored owing to local intervention without PD (transarterial chemoembolization [TACE], n = 1; radiofrequency ablation, n = 1; and liver transplantation, n = 3). The radiologic, ADC, and pathologic responses of the liver explants are detailed on Table 3. In contrast to 1 lesion with no obvious ADC change and poor pathologic response, favorable outcomes (near-total necrosis) were achieved in 2 lesions with high ADC changes (35% and 70%) post-SABR.

**Table 3:** Radiologic, ADC and pathologic responses of the liver explants.

	Tumor size (cm)	dose regimen	SABR-transplant interval (m)	1^st^ MRI response	ADC change	Pathology
Lesion 1	1.2	40 Gy/5fx	5.8	SD	−3%	No obvious necrosis
Lesion 2	1.7	50 Gy/5fx	8.5	CR	35%	95% necrosis
Lesion 3	5.7	45 Gy/5fx	7.3	CR	70%	100% necrosis

*Abbreviation:* ADC = apparent diffusion coefficient; fx = fraction; SABR = stereotactic ablative radiotherapy; SD = stable disease; CR = complete response.

## Discussion

Identifying early response to therapy allows for individualized treatment plans and potentially improves overall prognosis. Our results suggest that ADC change on early MRI is closely related to durable tumor control in HCCs treated with SABR.

Current enhancement-based criteria are highly predictive of HCC patient outcomes after ablation or embolization^7^. Nevertheless, the predictive values of these criteria have not been validated in the context of radiotherapy. In fact, the unique post-SABR HCC imaging features may preclude the interpretation of response based on current criteria^15,16^. A recent study by Mendiratta-Lala *et al*. focusing on MRI followed 67 HCCs following SABR; 58% had persistent arterial hyperenhancement and 54% had a washout appearance by 3–6 months^15^. These features disappeared over time without detectable tumor progression, resulting in alternating response rates (3–6 months: 25%; and 12 months: 70%). They concluded persistent arterial hyperenhancement is common and does not necessarily indicate viable tumor. Other SABR studies produced similar findings^16,17^. A retrospective study by Mannina *et al*. demonstrated poor concordance between pathologic response with available radiologic grading criteria^18^. Assessment based on mRECIST had a poor kappa coefficient of 0.224. In our study, the response rate on first MRI was 55.6%, which improved to 75.6% at the most recent follow-up. More than half of the lesions initially deemed to be SD had absent or declining arterial enhancement over time. Initial treatment response by mRECIST was not significantly correlated with long-term tumor control (*p* = 0.130). Collectively, these findings stressed that traditional response assessment criteria designed for HCC after locoregional therapy may not be useful post-SABR, particularly in early phases. The underestimation of the true effect of SABR may result in improper salvage treatment in clinical practice

DW MRI is a promising technique for early assessment of treatment response. The vast majority of HCC data pertain to TACE^19,20^ or transarterial radioembolization^21–23^. While anatomic changes post-locoregional therapies are noted within 1–3 months, a significant increase in ADC can be detected within several hours to 2 weeks post-therapy^19–23^. A pilot study by Eccles *et al*. evaluated ADC values in 4 HCCs, 2 cholangiocarcinomas, and 5 liver metastases post-SABR^14^ and found significant changes as early as 1 week into radiotherapy. Early ADC response was correlated with a higher dose and sustained tumor response, whereas no significant change in tumor size as assessed on T2 MRI at the same ADC time points were observed. Furthermore, Yu *et al*. demonstrated the added value of DW MRI for radiotherapy response prediction in 48 HCC patients treated with either hypofractionated radiotherapy (the majority) or SABR who had follow-up MRI at 3–5 months^13^. They found that mRECIST was a significantly better predictor of local progression-free survival (LPFS) than RECIST and an ADC change ≥20%. The latter 2 parameters combined predicted LPFS with comparable performance to the mRECIST. Another recent study that investigated imaging changes observed on MRI before and within 3 months of radiotherapy (3.5–5 Gy for 10 fractions) demonstrated that the ADC value increased in both responding and non-responding lesions with no significant difference (46.7% vs. 21.9%, *p* = 0.220)^24^.

In our study, we found that an ADC change of ≥25% within 6 months post-SABR was an independent predictor of sustained HCC tumor control, while the mRECIST was not. Our findings suggest a promising role for ADC in predicting early response in HCC patients treated with SABR. Different evaluation time points and methodologies may account, at least partly, for the divergence of our results from those of others.

In contrast to other publications on liver cancer radiotherapy response^13,14^, a relatively low *b* value of 500 s/mm^2^ was adopted in this study. Of note, the diffusion contrast and signal-to-noise ratio (SNR) should be simultaneously considered in DW MRI with different *b* values^25,26^. The better diffusion contrast in higher *b*-value images, and the more substantially reduced SNR are noted due to the increased MR signal loss. Several studies^26–28^ have reported that high *b* values (>500 s/mm^2^) are preferable in order to minimize perfusion effects, and a *b* value ranging between 500 and 800 s/mm^2^ is recommended for assessment of early response of HCC to nonsurgical local treatment. Because of the retrospective nature of the study, we did not have ADC data using a variety of *b* values. It was believed that the different *b* values may yield different ADC values, but the tendency of an ADC change ≥25% to indicate SABR in-field tumor control would not be changed.

Given the lack of universal standards, response assessment based solely on ADC change is evolving and requires further validation. DW MRI should be considered a routine adjunct modality to assess HCC after SABR with current enhancement-based criteria^29^. In our study, SD lesions were subdivided into DW(+) SD or DW(−) SD groups based on ADC response; with similar IFC rates following SABR, DW(+) SD lesions could receive sustained follow-up in line with mRECIST responding lesions. Early salvage treatment may be considered for DW(−) SD lesions because of their inferior IFC, as revealed in our study.

Our study had several limitations. First, the single-center, retrospective design and small sample size may have introduced selection bias. Second, the ADC values were not acquired at the same time point post-SABR; it may introduce bias in the judgement of ADC changes. Serial changes in ADC pre- and post-SABR should be investigated to determine the optimal protocol of DW MRI and accurately assess therapy response. Given most ADC values were obtained within 1–3 months post-SABR in this study, we believe the impact of different time intervals is relatively small. Third, there was no pathologic correlation between all treated lesions and their corresponding ADC change, although such validation by biopsy or surgery is not feasible for obvious ethical or technical reasons. Fourth, inter-observer difference for ADC measurement is an inevitable issue in clinical practice. Radiologists may subjectively choose different levels of slice and delineate diverse areas of ROI, even though the ROI measurement has been defined in this study. In order to minimize the variances, the final ADC value was the average of 3 ROIs obtained from 3 different slices, making our ADC change threshold more reliable.

In conclusion, we demonstrated that an ADC change ≥25% within 6 months is an independent predictor of sustained tumor control in HCC patients treated with SABR. Assessments based on mRECIST should be interpreted with caution, especially in the early phase post-SABR. Standardization of the appropriate DW MRI protocol and ADC acquisition parameters, as well as validation in prospective, large, multicenter trials, are warranted.

## Methods

### Data source

All HCC patients treated with SABR at our hospital between December 2007 and March 2018 were reviewed. The study eligibility criteria were (1) patients with available DW MRI sequences at baseline, and (2) patients with at least 1 DW MRI performed within 6 months post-SABR completion. HCC diagnosis was histologic or based on radiologic findings. Our institutional review board approved the study and waived the informed consent requirement owing to the investigation’s retrospective nature.

### MRI protocols and ADC measurement

MRI was performed on a 1.5-T MR system (Signa HDx, GE Healthcare) with an 8-channel body phased-array coil. The protocol of non-contrast and dynamic MR sequences have been detailed previously^30^. MRI protocol for liver tumors included non-fat suppressed axial and coronal single-shot fast spin-echo T2-weighted imaging (WI), axial fat suppressed fast spin echo T2WI, in- and out-of-phase, diffusion-weighted imaging and dynamic contrast enhanced T1WI with fat subtraction. A real time bolus-triggered technique using gadopentetate dimeglumine (Magnevist, Bayer HealthCare) was used to acquire the arterial (20–35 s), portal venous (70 s), and equilibrium phases (3 min).

DW MRI was performed in the axial plane with simultaneous use of respiratory triggering technique. DW images were obtained during dynamic MRI scanning; the TR was matched in each patient to the length of the respiratory cycle before gadoxetic acid enhancement. The scanning parameters were *b* values of 0 and 500 s/mm^2^; matrix size, 128 × 128; acceleration factor of SENSE, 4.0; field of view, 42 × 42 cm; number of excitations, 8; slice thickness, 6 mm; slice gap, 2 mm; and axial slices, 25. An ADC map using a monoexponential diffusion model was automatically generated.

Two radiologists (Wei-Chou Chang and Chih-Weim Hsiang) who were aware of the treated lesion but blinded to outcomes measured the ADC values. The DW MRI data were transferred to a separate computer-based workstation (GE Healthcare Systems) specifically designed for post-processing work, and the ADC measurement was calculated using a dedicated software tool (FuncTool, Advantage Workstation 4.3_07, GE Healthcare Medical Systems). Region of interests (ROIs) were delineated over the entire area of the treated lesion on the ADC image and positioned at identical or comparable slice positions on a T2-weighted image (the reference sequence). Each ADC measurement included 3 ROIs: the slice with the largest tumor diameter (the reference slice) and 2 other central slices between both ends and the reference slice. The final ADC value of each treated lesion was the average of those obtained from the 3 ROIs. If the inter-observer difference was larger than 15%, a third ADC measurement was conducted and the result was determined by consensus.

### SABR and follow-up

Treatment planning, dose prescription and radiation delivery methods have been detailed previously^31^. The SABR protocol is for both recurrent and newly-diagnosed HCC. In brief, patients were immobilized with a customized vacuum cushion during simulation and treatment. For most patients, fiducial markers in conjunction with a Synchrony respiratory motion-tracking system (Accuray Inc.) were employed to manage respiratory motion on a CyberKnife (Accuray Inc.). After June 2017, patients were treated with Versa HD (Elekta AB), using a controlled breath-hold technique or 4-dimensional computed tomography (CT) with forced abdominal compression to manage respiratory motion. The prescribed dose depended on the dose volume constraints of critical organs used in treatment planning^31^. The median prescribed does was 45 Gy (range, 30–60 Gy) in 4–6 fractions in 4–6 consecutive working days. Because of non-uniform fractionation, the dose regimens were converted to equivalent doses of 2 Gy per fraction with the assumption that the tumor α/β value was 10 Gy^32^.

Patients were assessed by clinical examination, blood work, and liver triphasic CT and/or MRI 1–3 months post-SABR completion and every 3–4 months thereafter. SABR response was assessed per tumor according to the mRECIST^5^. In-field failure was defined as presence of progressive disease (PD) or new enhancement within the planning target volume (PTV) or at its margins, which was defined as 1.5 cm from PTV, an area usually received high radiation dose.

### Statistical analysis

All analyses were performed on SPSS version 17 software (SPSS Inc.). Interobserver ADC value variability was evaluated using the intraclass correlation coefficient. The difference in ADC values pre- and post-SABR was evaluated using the Wilcoxon signed-rank test. The Mann-Whitney U-test was used to compare the differences in ADC values between SABR responding and non-responding lesions. In-field control (IFC) was calculated as the duration between initiation of SABR and in-field failure or the last follow-up. Data were censored at the time of liver transplantation or other local treatment involving the SABR-treated lesion in the absence of in-field failure. The Kaplan-Meier method was used to estimate IFC rates, with differences assessed using the log-rank test. Receiver operating characteristic (ROC) curves were used to establish the appropriate cut-off for predicting freedom from in-field failure. Variables with *p*-values < 0.1 on univariable analyses were subjected to multivariable analysis using a backward stepwise logistic regression model. A 2-tailed *p*-value < 0.05 was considered statistically significant in all analyses.

## Data Availability

The datasets generated during the current study are available from the corresponding author on reasonable request.
